# In Vitro Evaluation of DNA Damage Induction by Silver (Ag), Gold (Au), Silica (SiO_2_), and Aluminum Oxide (Al_2_O_3_) Nanoparticles in Human Peripheral Blood Mononuclear Cells

**DOI:** 10.3390/cimb46070417

**Published:** 2024-07-04

**Authors:** Milda Babonaitė, Emilija Striogaitė, Goda Grigorianaitė, Juozas Rimantas Lazutka

**Affiliations:** Institute of Biosciences, Life Science Center, Vilnius University, 7 Sauletekio Ave., LT-10257 Vilnius, Lithuania; emilija.striogaite@gmc.stud.vu.lt (E.S.); grigorianaitee@gmail.com (G.G.)

**Keywords:** nanoparticles, DNA damage, cytotoxicity, comet assay, reactive oxygen species

## Abstract

Nanoparticles (NPs) are increasingly applied in a wide range of technological and medical applications. While their use offers numerous benefits, it also raises concerns regarding their safety. Therefore, understanding their cytotoxic effects and DNA-damaging properties is crucial for ensuring the safe application of NPs. In this study, DNA-damaging properties of PVP-coated silver, silica, aluminum oxide (13 nm and 50 nm), and gold (5 nm and 40 nm) NPs in human peripheral blood mononuclear cells (PBMCs) were investigated. NPs‘ internalization and induction of reactive oxygen species were evaluated using flow cytometry. Cytotoxic properties were determined using a dual acridine orange/ethidium bromide staining technique while DNA-damaging properties were assessed using an alkaline comet assay. We observed that Ag, SiO_2_, and both sizes of Al_2_O_3_ NPs were efficiently internalized by human PBMCs, but only PVP-AgNPs (at 10–30 µg/mL) and SiO_2_ NPs (at concentrations > 100 µg/mL) induced significant DNA damage after a 24 h exposure. In contrast, the uptake of both sizes of gold nanoparticles was limited, though they were able to cause significant DNA damage after a 3 h exposure. These findings highlight the different responses of human PBMCs to various NPs, emphasizing the importance of their size, composition, and internalization rates in nanotoxicology testing.

## 1. Introduction

Nanotechnology is rapidly becoming one of the fastest-growing markets globally, significantly revolutionizing various industries [1]. In 2022, the global market for nanomaterials (NMs) was valued at USD 10.88 billion and is expected to grow at a compound annual growth rate (CAGR) of 14.8% from 2023 to 2030 [2]. The European Chemicals Agency (ECHA) anticipates that the EU’s NMs market will expand at a CAGR of 13.9% in volume and 18.4% in value over the next 5 years, creating a positive economic impact [3]. According to European Commission Recommendation 2022/C 229/01, nanomaterials are described as materials with one or more external dimensions in the size range of 1 nm–100 nm [4]. Nanoparticles (NPs) possess unique physicochemical properties due to their small size, composition, shape, and surface functionalities, enabling their use in various industries such as food science, cosmetics, pharmaceuticals, electronics, etc. [5,6,7,8].

Silver nanoparticles are widely used in biomedicine due to their antibacterial, antiviral, and antifungal properties against *Staphylococcus aureus* [9], *Candida albicans* [10], *Herpes simplex* virus (HSV), and human parainfluenza virus type 3 (HPIV-3) [11]. Currently, silver nanoparticles are included in many products, such as antibacterial dressings, home water treatment systems, cosmetics, and textiles [12]. In recent years, special attention has been paid to silica nanoparticles (SiO_2_) because of their wide range of applications in drug delivery, environmental remediation, and advanced catalysis [13], making them one of the most abundant nanoparticles on Earth [14]. Similarly, Al_2_O_3_ nanoparticles have been successfully used in drug delivery [15] and in ceramics to enhance the mechano-physical properties of ceramic tiles [16]. The unique chemical, physical, and photonic properties of gold nanoparticles have facilitated their use in medical biophysics [17], molecular imaging [18], and biosensors [19]. Gold nanoparticles tend to accumulate at tumor sites through a process known as the enhanced permeability and retention effect (EPR) [20]. When exposed to radiation, these NPs emit secondary electrons, which induce indirect DNA damage within cancer cells. Therefore, gold nanoparticles can be successfully used as radiosensitizers in cancer therapy [21]. Despite the numerous benefits of NPs in various industries, the exponential growth of nanotechnology has led to increased human exposure to nanomaterials, raising concerns about their safety.

Numerous studies have demonstrated the ability of NPs to accumulate in cells and organs, leading to the generation of reactive oxygen species (ROS) and genotoxic, DNA-damaging effects. Nanoparticles can induce DNA damage directly by binding to DNA or indirectly by interfering with nuclear proteins or generating oxidative stress [22]. However, the genotoxicity of various nanoparticles remains a controversial question. The DNA-damaging properties of AgNPs have been confirmed in vitro [23] and in vivo [24], while other studies have demonstrated no such effects [25,26,27]. Similarly, some studies have shown that the genotoxicity of AuNPs is size-dependent [25], whereas other studies have found that neither the size nor the functionalization of AuNPs accounts for their genotoxic effects [28]. For SiO_2_ and Al_2_O_3_ NPs, no significant DNA or chromosomal damage was observed in human lymphocytes in vitro [29], while Zhang et al. [30] demonstrated ROS generation and markers of genotoxicity in mice exposed to alumina NPs.

Because of the inconsistencies in previous studies regarding the DNA-damaging properties of various nanoparticles, the aim of this study was to evaluate the DNA-damaging properties of six nanoparticles: 35 nm PVP-coated silver (PVP-Ag) NPs, 10–20 nm silica (SiO_2_) NPs, aluminum oxide (Al_2_O_3_) NPs of two sizes (13 nm and 50 nm), and gold (Au) NPs of two sizes (5 nm and 40 nm) in human peripheral blood mononuclear cells (PBMCs). The selection of nanoparticles for our study was primarily based on their commercial availability and the established applications of different nanoparticle sizes. Gold nanoparticles are preferred to be as small as possible in biomedical applications such as imaging, therapy, and diagnostics due to their biocompatibility and unique optical properties [31,32]. Hence, we chose 5 nm nanoparticles for these reasons. To evaluate the impact of size on particle toxicity, we also included 40 nm gold nanoparticles. This larger size will help us understand any size-dependent differences in genotoxicity. A similar rationale was applied to the selection of Al_2_O_3_ NPs. Larger nanoparticles (50–150 nm) can be used in coatings, electronics, and ceramics [33,34], whereas smaller particles tend to be more reactive and are used in catalysis [35]. We chose both sizes to encompass many applications in the field. The predominant use of SiO_2_ nanoparticles is in drug delivery, where smaller sizes are preferred because of the easier loading and uptake of NPs [36,37]. Finally, for the silver nanoparticles, their reactivity and antibacterial properties are most effective at sizes up to 50 nm [38], therefore 35-nm-sized particles were selected. Overall, the selected nanoparticles possess unique properties that lead to widespread applications; therefore, evaluation of their safety is important. In this study, nanoparticle internalization was investigated using flow cytometry light scattering analysis, and the ability to induce reactive oxygen species (ROS) generation was analyzed using the H_2_DCFDA assay. To determine cytotoxicity, a dual acridine orange/ethidium bromide staining technique was applied, and DNA-damaging properties were assessed using an alkaline comet assay.

## 2. Materials and Methods

### 2.1. Chemicals and Reagents

This study tested the cytotoxic and genotoxic potential of six different nanoparticles. 35 nm PVP-coated AgNPs were kindly provided by UAB Rho Nano (Vilnius, Lithuania); 10–20 nm SiO_2_ (CAS No. 7631-86-9, Cat. No. 637238), Al_2_O_3_ 13 nm (CAS No. 1344-28-1, Cat. No. 718475), Al_2_O_3_ 50 nm (CAS No. 1344-28-1, Cat. No. 544833), Au 5 nm (Cat. No. 741949) and Au 40 nm (Cat. No. 753637) were purchased from Sigma Aldrich (St. Louis, MO, USA). After purchase, the nanoparticles were stored according to the manufacturers‘ recommendations.

DMSO was obtained from Merck KGaA (Darmstadt, Germany). Ethidium bromide, low melting point agarose (LMP), Na_2_EDTA, Tris HCl, and Triton X-100 were obtained from Carl Roth GmbH (Karlsruhe, Germany). Phosphate-buffered saline (PBS, Ca^2+^, and Mg^2+^ free) was purchased from Gibco (New York, NY, USA). All other chemicals were obtained from Sigma Chemical Co. (St. Louis, MO, USA).

### 2.2. Preparation of NP Suspensions and Particle Characterization

Suspensions of nanoparticles were prepared at a concentration of 0.1% (1 mg/mL). AgNP suspension was prepared in 0.2% PVP solution. SiO_2_, Al_2_O_3_ 13 nm, and Al_2_O_3_ 50 nm suspensions were prepared in cell culture media—RPMI 1640. Au 5 nm and Au 40 nm were supplied as a colloidal stabilized stock suspension in citrate buffer. Nanoparticle solutions were sonicated at 35 kHz for 30 min in a Bandelin Sonorex Super sonication bath (BANDELIN electronic GmbH & Co. KG, Berlin, Germany) and immediately used in the uptake, ROS induction, cytotoxicity, and genotoxicity studies.

Hydrodynamic particle size was evaluated via Nanoparticle Tracking Analysis (NTA) (Nanosight LM10, Malvern Panalytical Ltd., Malvern, UK) immediately (0 h), 1 or 3, and 24 h after sonication. The samples were injected into the chamber with a sterile syringe until the liquid reached the tip of the nozzle. Each measurement was performed at 22 ℃, with a camera level of 10. The Nanosight NTA 3.1 analytical software was employed. The highest peak size (size distribution peak with most particles) and mean particle size distribution were determined by tracking analysis of the particles’ Brownian motion in solution [39].

### 2.3. Biological Material

Experiments were conducted using peripheral blood mononuclear cells (PBMCs) obtained from healthy 22–36-year-old volunteers (non-smoking, with no known illness). Peripheral blood was collected by venipuncture in heparinized vacutainer tubes (Becton-Dickinson, Franklin Lakes, NJ, USA). Informed consent was obtained from all subjects involved in the study. The study was conducted in compliance with research ethics requirements adopted by Vilnius University and approved by the Doctoral Committee of Vilnius University (Authorization No. 93 (21 November 2019).

PBMCs were isolated using Lymphropep™ density gradient centrifugation according to the manufacturer’s instructions (Axis-Shield, Oslo, Norway). Equal parts of the blood and RPMI 1640 cell culture medium were added to a centrifuge tube, followed by stratification with an equal part of Lymphoprep™ and centrifugation of the solution at room temperature at 800× *g* for 20 min. Subsequently, the mononuclear cell layer was carefully aspirated and washed with RPMI 1640 medium by centrifugation at 800× *g* for 10 min.

### 2.4. Cellular Uptake Analysis using Flow Cytometry

To determine the potential uptake of NPs in PBMCs, flow cytometry light scatter analysis was conducted according to Suzuki et al. [40]. Following PBMC isolation, cells were resuspended in RPMI 1640 medium at a concentration of 4 × 10^5^. The cells were then transferred into sterile 15 mL tubes and exposed to various concentrations of different NPs for 24 h (0–160 µg/mL of SiO_2_ and Al_2_O_3_ NPs; 0–50 µg/mL of AgNPs; 0–7.5 µg/mL of Au 5 nm; 0–4.5 µg/mL of Au 40 nm). After exposure to NPs, the cells were centrifuged at 800× *g* for 10 min, the supernatant was removed, and the cells were resuspended in phosphate-buffered saline (PBS). Subsequently, NP uptake was evaluated using flow cytometric light scatter analysis. Ten thousand cells were measured in each sample using a FACSCalibur (BD Biosciences) flow cytometer, and data analysis was performed using Floreada.io software (https://floreada.io, accessed on 5 April 2024). The intensities of forward-scatter(ed) (FSC) light, which represents cell size, and side-scatter(ed) light (SSC), which is proportional to intracellular density and granularity that reflects NP uptake, were measured [40,41].

### 2.5. Intracellular ROS Evaluation

Reactive oxygen species (ROS) production in human PBMCs was evaluated using the cell-permeant 2′,7′-dichlorodihydrofluorescein diacetate (H_2_DCF-DA) fluorescent probe (Abcam, Cambridge, UK). Intracellular ROS induces the conversion of H_2_DCF-DA into a cell-impermeable green fluorescent product that can be quantified by flow cytometry within the FITC channel (excitation: 488 nm/emission: 519 nm) [42].

For this analysis, PBMCs were prepared exactly as for the cellular uptake analysis but after centrifugation, the cells were stained in cell culture media with 20 µM H_2_DCF-DA. Samples were incubated for 30 min at 37 °C and immediately analyzed on a flow cytometer. Ten thousand cells were analyzed for each sample, and data processing was performed using Floreada.io software.

### 2.6. Cell Viability and DNA Damage Analysis

Once the uptake of NPs and ROS generation were evaluated, the cytotoxicity and DNA damage-inducing potential of tested NPs were assessed. Following PBMC isolation, cells were resuspended in RPMI 1640 medium at 1–2 × 10^5^ cells/mL in sterile 15 mL centrifuge tubes and treated with different concentrations of NPs for 1 or 3 and 24 h at 37 °C in a 5% CO_2_ environment. As a positive control, 20 μM hydrogen peroxide was used, 1 h before the end of incubation time. A negative/untreated (0 µg/mL) control was also included. After the exposure, samples were centrifuged at 800× *g* for 10 min. The supernatant was removed and the cells were resuspended in RPMI 1640 medium.

Cytotoxicity was determined by calculating the number of viable cells using a dual acridine orange and ethidium bromide (AO/EB) staining technique, according to Liu et al. [43] with minor modifications, as previously described. Briefly, the staining solution was prepared by combining 1 µL of AO (5 mg/mL) and 1 µL of EB (3 mg/mL) with 1 mL PBS. Finally, 20 µL of cell suspension and 2 µL of prepared AO/EB stain were placed on a clean microscope slide, covered with a cover slip, and analyzed under a fluorescent microscope (Nikon Eclipse 80*i*, Fujisawa, Japan). At least 100 cells from each sample were scored to determine the percentage of viable cells. According to Azqueta et al. [44], high levels of cytotoxicity can influence DNA migration in the comet assay. To minimize the risk of false positive results, it is recommended that cell viability be maintained above 70–75%.

Levels of primary DNA damage were determined using an alkaline comet assay according to Singh et al. [45], with slight modifications exactly as previously described [46]. Briefly, 40 µL of cell suspension was mixed with 40 µL of fresh 1% low melting point agarose (LMP) in PBS at 37 °C (final LMP concentration—0.5%). A mixture of cells and agarose (80 µL) was pipetted onto glass microscope slides precoated with 1% normal melting point (NMP) agarose and covered with a 24 mm × 24 mm coverslip and allowed to solidify for 10 min at 4 °C. Two gels were prepared per sample. After the gels solidified, the coverslips were gently removed and the slides were placed in a cold freshly prepared lysis solution (2.5 M NaCl, 100 mM Na_2_EDTA, 10 mM Tris, with 1% Triton X-100 and 10% DMSO added just before use, pH 10) and kept in the dark for 90 min at 4 °C. After lysis, slides were placed in a horizontal gel electrophoresis tank COMET-20 SYSTEM (Scie-Plas, Cambridge, UK) filled with cold (4 °C), fresh electrophoresis buffer (1 mM Na_2_EDTA and 300 mM NaOH, pH 13) and left in the solution for 20 min to facilitate DNA unwinding. Then, electrophoresis was carried out at 19 V and 300 mA (1 V/cm) for 30 min. To maintain the buffer temperature during electrophoresis, the platform was cooled using a refrigeration unit (FL300, Julabo, Seelbach, Germany), and the circulation of the buffer was additionally maintained by a pump (Watson-Marlow sci Q400, Marlow, UK). After electrophoresis, the slides were neutralized with Tris HCl buffer (0.4 M Tris HCl, pH 7.5) and each gel was stained with 80 µL of 20 µg/mL ethidium bromide. All the above steps were conducted under dim light to prevent additional DNA damage.

The slides were examined under 400× magnification using a fluorescence microscope (Nikon Eclipse 80*i*, Japan) by a single scorer. Image capture and analysis were performed using LUCIA Comet Assay™ software version 7.60 (Laboratory Imaging, s.r.o., Praha, Czech Republic). For each sample, two gels were prepared and 50 nucleoids (“comets”) per gel were randomly selected and scored, resulting in a total of 100 comets per sample. The comet’s head contains intact DNA, while fragmented (damaged) DNA is located in its tail. Thus, the percentage of DNA in the comet tail (% TDNA) was used as an indicator of DNA damage.

Five independent cytotoxicity and comet assay experiments using blood samples from different donors were carried out. Results are presented as mean ± SEM. The statistical significance of the results was assessed using Student’s *t*-test. To describe the relationship between NP concentrations and their effects on PBMCs, a linear regression model was applied.

## 3. Results

### 3.1. Characterization of the Hydrodynamic Diameter of Nanoparticles

The hydrodynamic diameter of nanoparticles in cell culture medium (RPMI 1640) was evaluated using NTA immediately (0 h), 1 or 3 h (depending on NPs tested), and 24 h after sonication (Table 1). The NTA analysis showed that all tested nanoparticles agglomerated in cell culture medium in time, as mean size distributions of up to 320 nm were observed.

Nanoparticles with a primary size of less than 20 nm, such as SiO_2_ (10–20 nm), Al_2_O_3_ (13 nm), and Au (5 nm), exhibited significant agglomeration in cell culture media, with mean particle sizes increasing up to 20–30 times their primary times. It is important to note that it may be difficult to detect primary nanoparticles that are approximately 10 nm in size, as they can be overshadowed by the larger agglomerates [25]. In comparison, Al_2_O_3_ 50 nm and Au 40 nm retained some fraction of their primary size (73–82 nm), with the mean particle sizes being 3–4 times larger than their primary sizes.

### 3.2. Nanoparticle Uptake Analysis

**Figure 1:**
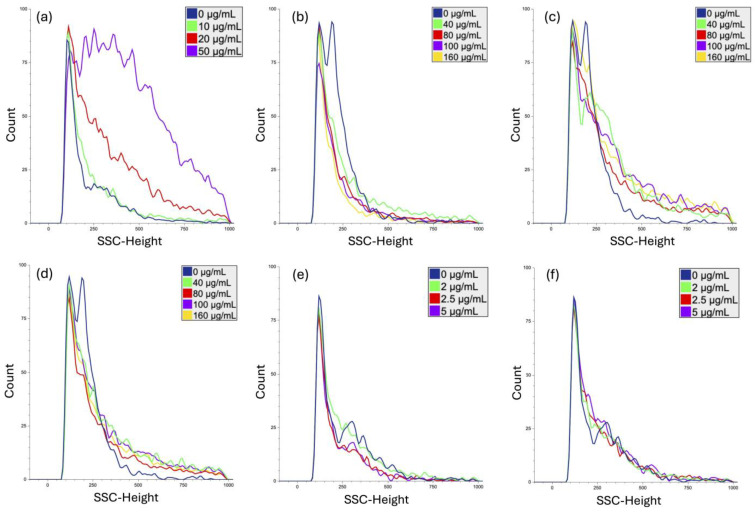
Uptake of PVP-Ag (**a**), SiO_2_ (**b**), Al_2_O_3_ 13 nm (**c**), Al_2_O_3_ 50 nm (**d**), Au 5 nm (**e**), and Au 40 nm (**f**) nanoparticles by human PBMCs, following a 24 h exposure, analyzed using flow cytometry. Control cells (0 µg/mL) were cultivated in NP-free cell culture media.

The capacity of tested NPs to be internalized by human PBMCs, following 24 h of exposure, was evaluated by measuring changes in side-scattering light intensities using a flow cytometer (Figure 1). The percentage of relative uptake (fold-increase) was determined by measuring the increase in the side-scattering light intensities compared to the untreated control.

PVP-coated Ag nanoparticles were efficiently taken up by human PBMCs. Compared to the untreated control, nearly 1.2-fold, 1.6-fold, and 2.8-fold increases in SSC intensities were observed when cells were exposed to 10, 20, and 50 µg/mL of AgNPs, respectively, (Figure 1a).

A 2.2–2.4-fold increase in SSC intensities over the background was detected following human PBMC exposure to different concentrations of SiO_2_ (40–100 µg/mL), indicating the notable uptake of these nanoparticles (Figure 1b). Although uptake decreased at 160 µg/mL, likely due to the formation of larger agglomerates, SSC intensities remained 1.6-fold higher than in the untreated control.

Even higher SSC intensities (histogram shifts to the right) were observed following a 24 h treatment of human PBMCs to Al_2_O_3_ 13 nm and 50 nm NPs (40–160 µg/mL) (Figure 1c,d, respectively). Compared to the untreated control, 1.9-fold, 2.8-fold, 2.5-fold, and 3-fold increases were detected at 40, 80, 100, and 160 µg/mL concentrations of Al_2_O_3_ 13 nm NPs (Figure 1c), while 3-fold, 2.8-fold, 3.5-fold, and 3.6-fold increases in SSC intensities were observed in PBMCs exposed to 50 nm Al_2_O_3_ NPs, respectively, (Figure 1d). Although the uptake of larger Al_2_O_3_ nanoparticles was more efficient, the uptake of smaller nanoparticles was concentration-dependent (R^2^ = 0.85, *p* = 0.026).

In contrast, following 24 h exposure of human PBMCS to different concentrations (2–4.5 µg/mL) of 5 and 40 nm gold nanoparticles, no changes in SSC intensities were observed, indicating limited internalization (Figure 1e,f, respectively).

### 3.3. Generation of Reactive Oxygen Species

**Figure 2:**
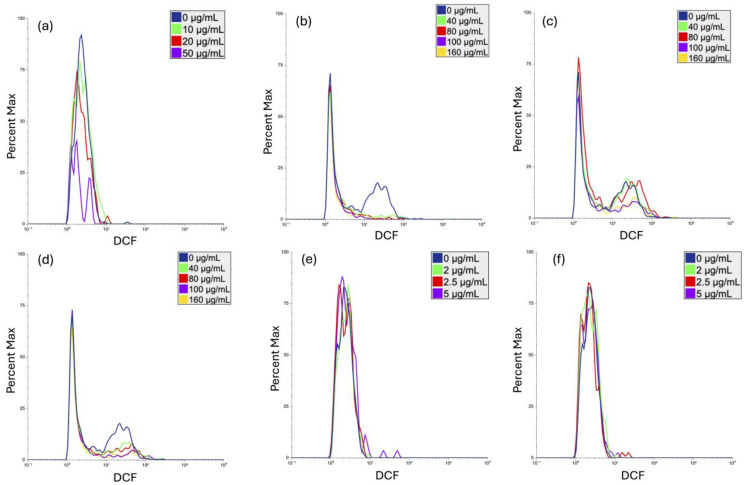
Generation of reactive oxygen species (ROS) in human PBMCs following 24 h of exposure to Ag-PVP (**a**), SiO_2_ (**b**), Al_2_O_3_ 13 nm (**c**), Al_2_O_3_ 50 nm (**d**), Au 5 nm (**e**), and Au 40 nm (**f**) nanoparticles was determined using H_2_DCFDA assay and flow cytometry. Control cells (0 µg/mL) were cultivated in NP-free cell culture media.

One of the pathways through which nanoparticles induce DNA damage is via ROS generation [47]. Therefore, the nanoparticles‘ abilities to induce reactive oxygen species (ROS) generation in human PBMCs after a 24 h exposure was evaluated using H_2_DCFDA fluorescent dye and a flow cytometer (Figure 2).

Most of the particles tested did not induce ROS generation. No significant induction of ROS in human PBMCs, following 24 h exposure to PVP-coated Ag nanoparticles (10–50 µg/mL) was observed compared to the untreated control (0 µg/mL) (Figure 2a). Similarly, ROS generation was not induced by SiO_2_ NPs and AuNPs, regardless of their size (5 or 40 nm) (Figure 2b and Figure 2e,f, respectively).

Interestingly, aluminum oxide nanoparticles induced slight ROS generation in human PBMCs compared to the background levels (7.1%). The highest levels of ROS were generated in cells exposed to 160 µg/mL of 13 nm Al_2_O_3_ NPs and to 100 µg/mL of 50 nm Al_2_O_3_ NPs (8.2% and 10.0%, respectively; Figure 2c,d). Compared to the untreated control, these ROS changes were not considered biologically relevant; however, they should be taken into consideration.

### 3.4. Cytotoxicity and Induction of DNA Damage

Human PBMCs were treated with different concentrations of nanoparticles (Ag, SiO_2_, Al_2_O_3_, and Au) for 1 or 3 and 24 h to determine their cytotoxic potentials and DNA-damaging properties. DNA damage was assessed using an alkaline comet assay and simultaneously, cytotoxicity was evaluated by counting cell viability using a dual ethidium bromide and acridine orange staining technique.

**Figure 3:**
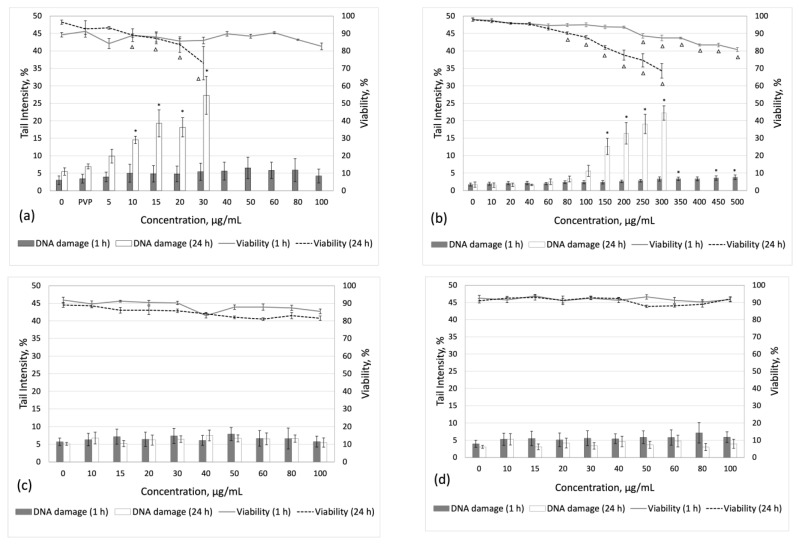

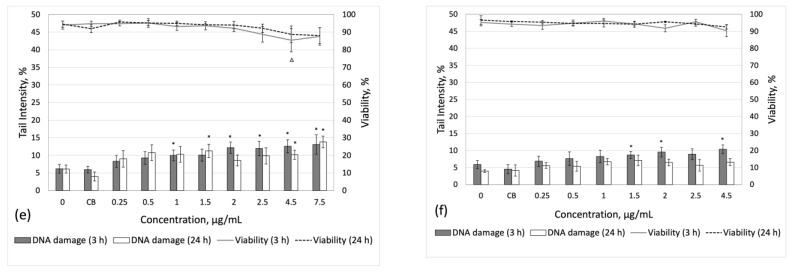
Human PBMC viability and induced DNA damage following short-term (1 or 3 h) and long-term (24 h) exposure to (**a**) Ag-PVP, (**b**) SiO_2_, (**c**) Al_2_O_3_ 13 nm, (**d**) Al_2_O_3_ 50 nm, (**e**) Au 5 nm, and (**f**) Au 40 nm nanoparticles. Data are presented as mean values of five donors. Results are considered statistically significant (* or Δ) when *p*  ≤ 0.05. PVP—0.2% polyvinilpyrolidone solution; SC—solvent control (citrate buffer).

Human PBMCs exposed to PVP-coated Ag nanoparticles at 5–100 µg/mL showed no relevant cytotoxic responses after a 1 h exposure (Figure 3a). However, after a 24 h treatment, concentrations higher than 30 µg/mL reduced cell viability by more than 40% and were therefore considered cytotoxic and not tested further. No significant induction of DNA damage was observed following a 1 h exposure to AgNPs, whereas after 24 h of exposure, human PBMCs showed a more than 2-fold increase in DNA damage at 10–20 µg/mL, which then increased to 3.9-fold at 30 µg/mL over the solvent control (0.2% PVP in water) (Figure 3a).

SiO_2_ NPs tested at 10–500 µg/mL showed a concentration-dependent decrease in cell viability after a 1 h exposure, with a minimum viability value of 80.8% (500 µg/mL) (Figure 3b). After long-term (24 h) exposure, a statistically significant reduction in cell viability was observed at 80–300 µg/mL, while concentrations higher than 300 µg/mL reduced cell viability by more than 40% and were therefore considered cytotoxic and not tested further. Following a 1 h exposure, SiO_2_ NPs (0–500 µg/mL) did not induce significant amounts of DNA damage. However, after long-term treatment, a significant concentration-dependent increase in DNA strand breaks was observed (R^2^ = 0.96, *p* < 0.001). At the highest tested concentration of 300 µg/mL, SiO_2_ NPs induced a 13-fold increase in DNA damage compared to the untreated control (Figure 3b).

Al_2_O_3_ nanoparticles did not exert any relevant cytotoxicity, regardless of their size (13 or 50 nm) and exposure time (1 or 24 h) (Figure 3c,d). Furthermore, no statistically significant increase in DNA damage was observed compared to the untreated control.

No reduction in cell viability was observed when human PBMCs were exposed to 5 nm AuNPs of up to 7.5 µg/mL, and 40 nm NPs of up to 4.5 µg/mL (the highest test concentrations possible from the supplied stock sample), regardless of the exposure time (3 or 24 h) (Figure 3e,f). After short-term (3 h) exposure, 5 nm NPs significantly increased DNA damage at 1, 2, 2.5, 4.5, and 7.5 µg/mL in a concentration-dependent manner (R^2^ = 0.63, *p* = 0.01) (Figure 3e). Similarly, 40 nm AuNPs induced significant concentration-dependent DNA damage at 1.5, 2, 4.5 µg/mL (R^2^ = 0.7, *p* = 0.009) (Figure 3f). Interestingly, 40 nm AuNPs had no significant effect on human PBMCs after 24 h of exposure (Figure 3f), while 5 nm NPs continued to show significant DNA damage at 1.5, 4.5, and 7.5 µg/mL (Figure 3e). Overall, smaller nanoparticles, with a primary size of 5 nm, induced more DNA damage compared to particles with a primary size of 40 nm.

## 4. Discussion

This study investigated the cytotoxic effects and DNA-damaging properties of SiO_2_, PVP-coated Ag, Al_2_O_3_, and AuNPs in human peripheral blood mononuclear cells using dual acridine orange/ethidium bromide staining technique for cell viability evaluation and an alkaline comet assay for DNA damage analysis. The uptake of tested nanoparticles and ROS generation was monitored using flow cytometry.

Nanoparticle tracking analysis (NTA) revealed that all tested nanoparticles agglomerated in cell culture media, in most cases resulting in sizes larger than their primary sizes. Regarding the impact of nanoparticle agglomeration on their toxicity, there was no consensus. Murugadoss et al. [48] investigated the toxicity of small agglomerates (SAs) and large agglomerates (LAs) of TiO_2_ nanoparticles. The study revealed that in most in vitro analyses, there were no significant differences between SA and LA samples, leading to the conclusion that LAs are not less active than SAs. Interestingly, notable differences were observed in THP-1 cells, where LAs induced more damage than SAs. THP-1 cells, being phagocytic, may be more suitable for the uptake of submicron and micron-sized agglomerates, resulting in higher LA uptake and increased cellular damage compared to SAs. In our study, the peripheral blood mononuclear cell layer mainly contains lymphocytes, with a small number of monocytes [49], which could explain the slightly higher uptake levels of larger agglomerates (SiO_2_, Al_2_O_3_ NPs, etc.) compared to smaller ones (AuNPs). However, it is important to note that the DNA-damaging potential is influenced not only by the agglomeration or uptake of NPs but also by the composition of particles and selected cell lines. Magdolenova et al. [50] proposed that larger agglomerates might be less stable, allowing individual NPs to be released from the agglomerate and subsequently taken up by the cells. They also suggested that larger agglomerates precipitate quickly, potentially leading to higher real exposure to NPs compared to particles dispersed in the cell culture media, thus making them more toxic. Their study showed that large agglomerates induced more DNA damage in all tested cell cultures in vitro, whereas NP suspensions with agglomerates smaller than 200 nm had no genotoxic effects. Overall, there are mixed opinions on whether agglomeration increases the toxicity of nanoparticles. We believe that while agglomeration can facilitate the uptake of NPs in some cases, toxicity is influenced by multiple factors beyond agglomeration alone.

Following 24 h exposure, PVP-coated 35 nm Ag nanoparticles were taken up by human PBMCs in a dose-dependent manner, causing significant amounts of DNA damage even at low concentrations (10–30 µg/mL) without inducing ROS. Other researchers have also demonstrated efficient cellular uptake of PVP-coated AgNPs by human PBMCs [23,51,52]. Vukovic et al. showed a dose-dependent uptake of PVP-coated AgNPs (with a primary size of 10.7 nm) by human PBMCs after 1 and 3 h of exposure, with significant DNA damage observed at 1 µg/mL [23]. Their study also revealed that PVP-AgNPs significantly increased ROS levels after 1 h of exposure, as detected by the H_2_DCFDA assay, but not after 3 h. The absence of significant ROS levels at later time points, such as 3 or 24 h (as in our study) can be attributed to the effective neutralization of ROS by cellular antioxidant defenses [53]. However, other ROS detection methods, such as DHE assay and DiOC_6_ staining, showed a significant oxidative stress response in human PBMCs exposed to PVP-AgNPs [23], highlighting that the sensitivity and specificity of the ROS detection methods can register different types of ROS and yield varying results. In contrast, Folbjerg et al. showed a substantial increase in ROS production in THP-1 monocytes following 6 and 24 h of exposure to 69 nm PVP-coated AgNPs using the H_2_DCFDA assay [54]. Monocytes, known for their role in directly combating pathogens through oxidative mechanisms, are prolific ROS producers, which may explain the discrepancy between their findings and ours, where lymphocytes predominate due to naturally lower levels of monocytes in the blood [55]. Additionally, DNA damage induced by PVP-AgNPs was confirmed in the human lung epithelial cells, BEAS-2B and A549 [56,57], while PVA-coated AgNPs induced DNA damage in HepG2 cells and human PBMCs [58]. Conversely, PVP-coated 50 and 200 nm size nanosilver did not exhibit any genotoxic effects in 3D human bronchial models [59]. Compared to 2D models, the uptake of NPs is much lower in 3D models, which may result in lower levels of DNA damage in 3D models.

SiO_2_ NPs were efficiently internalized by human PBMCs following 24 h of exposure, resulting in significant DNA strand breaks, but only at high concentrations (150–300 µg/mL). No significant DNA damage was detected at lower concentrations (10–100 µg/mL) regardless of the exposure time (1 or 24 h). Furthermore, no significant ROS generation was observed at the tested concentrations (0–100 µg/mL). In a study by Gonzalez et al. [60], the genotoxicity of 16 nm and 60 nm SiO_2_ nanoparticles was evaluated in the A549 cell line after short-term exposure (15 min and 4 h). Similar to our findings, no significant increase in DNA strand breaks or oxidative damage was observed regardless of the particle size and exposure time. In contrast, unmodified 70 nm SiO_2_ nanoparticles induced intracellular ROS generation in HaCaT and TLR-1 cells in a dose-dependent manner, with significant DNA strand breaks observed at 90 µg/mL in HaCaT cells [61]. Additionally, after 24 h of exposure to 14 nm SiO_2_ NPs, cellular uptake by A549 cells was confirmed and DNA damage was evident at 0.1 µg/mL in A549, HT29, and HaCaT cells [62]. However, in a study involving human PBMCs, it was determined that although 10–50 nm size SiO_2_-NPs can be efficiently internalized by human PBMCs, no cytotoxicity or genotoxicity was detected at 100 µg/mL, regardless of the exposure time (2 or 24 h).

Our study confirmed the intracellular uptake of Al_2_O_3_ nanoparticles, with the 50 nm particles being internalized more efficiently than particles with a primary size of 13 nm. It is known that NP uptake strongly depends on their size [63]. Several studies have indicated that the optimal size for efficient uptake is approximately 50 nm [64,65,66]; however, other properties, including shape, composition, and surface charge of NPs have to be taken into consideration as well [63]. A slight increase in ROS was observed following 24 h of exposure to Al_2_O_3_ NPs. However, no significant DNA damage was determined, regardless of nanoparticle size or exposure time. Our results are in agreement with data from a study by Jallili et al. [67], where the internalization of Al_2_O_3_ NPs with a primary size of 30 nm by Caco-2 and HepaRG cells was confirmed but no genotoxic activity was observed in the γH2AX assay as well as the comet assay. Although no significant increase in DNA damage evaluated by the comet assay was observed in human PBMCs and HEK293 cells by other researchers [29,68], in a study by Sliwinska et al. [69], concentration-dependent genotoxic effects of 30 nm Al_2_O_3_ NPs after 24 h exposure were determined in human PBMCs. However, the characterization of NPs or cellular internalization was not investigated in their study, which may explain the discrepancy in the responses.

Interestingly, the uptake of 5 nm and 40 nm AuNPs by human PBMCs was limited. Despite the fact that no ROS was generated, both AuNPs induced significant DNA damage in a concentration-dependent manner following a 3 h exposure. However, after 24 h of exposure, only particles with a primary size of 5 nm (at 1.5, 4.5, and 7.5 µg/mL) were able to induce significant DNA damage. Overall, smaller nanoparticles were more genotoxic in human PBMCs compared to particles with a primary size of 40 nm. Other researchers have also demonstrated that the particle size of AuNPs affects their genotoxicity. In a study by Lebedova et al., 5 nm AuNPs induced more DNA damage compared to 50 nm particles in normal human bronchial epithelial cells [25]. Similarly, Xia et al. showed that 5 nm AuNPs induced a dose-dependent increase in DNA damage in the HepG2 cell line, while the 20 and 50 nm particles did not [70]. Generally, smaller nanoparticles have enhanced surface area, exposed surface atom ratio, and elevated catalytic capabilities, resulting in higher toxicity [71]. It was suggested that smaller gold nanoparticles release toxic ions and inhibit thioredoxin reductase, damaging mitochondria and inducing secondary DNA damage [72]. Interestingly, May et al. revealed that AuNPs (2–4 nm) were efficiently internalized by A549 cells without inducing inflammatory reactions [73]. They demonstrated that after a 3 h exposure to AuNPs, no significant DNA damage was induced. However, DNA damage increased significantly following 24 h treatment at 40–100 µg/mL. Concentrations comparable to those used in our study (up to 10 µg/mL) showed no genotoxic effects, regardless of the exposure time [73]. However, according to Paino et al. [74], 18.2 nm citrate-coated AuNPs (at 1 and 50 µM) induced significant ROS generation in human PBMCs and HepG2 cells, but no significant DNA damage was revealed, except at 50 µM in HepG2 cells, as detected by the comet assay. This suggests that HepG2 cells may be more sensitive to the DNA-damaging action of AuNPs compared to human PBMCs. Overall, variability parameters, such as the cell lines used, exposure time, concentrations, surface charge, and coating of nanoparticles can have a major impact on their genotoxic effects.

## 5. Conclusions

Altogether, our results demonstrated that the majority of tested nanoparticles (PVP-Ag, SiO_2_, and Al_2_O_3_) were efficiently internalized by human PBMCs. Following a 24 h exposure, PVP-Ag nanoparticles induced significant amounts of DNA damage at 10–30 µg/mL, while DNA-damaging properties of SiO_2_ NPs were demonstrated only at higher concentrations exceeding 100 µg/mL. Interestingly, internalization of AuNPs by human PBMCs was not observed, regardless of particle size, but a significant amount of DNA damage was demonstrated after a 3 h exposure. Additionally, it was shown that the DNA-damaging potential of Al_2_O_3_ and Au nanoparticles was size-dependent, with smaller nanoparticles (13 nm and 5 nm, respectively), inducing more DNA damage compared to 50 nm Al_2_O_3_ and 40 nm AuNPs_._ Overall, nanoparticles exhibit varying mechanisms of DNA damage. Some NPs are efficiently internalized by human PBMCs and can induce DNA damage, while others are taken up by the cells without causing DNA damage or ROS generation. In contrast, AuNPs were not internalized by the cells but were shown to cause significant DNA damage, likely through indirect mechanisms.

## Figures and Tables

**Table 1:** Hydrodynamic size distributions of Ag, SiO_2_, Al_2_O_3_, and AuNPs in RPMI 1640 medium 0, 1, or 3, and 24 h after sonication. Highest peak size—size distribution peak with most particles, SD—standard deviation calculated using the NTA software.

Nanoparticles and Their Primary Sizes	Time after Sonication, h	Highest Peak Size, nm	Mean Size, nm (SD)
Ag 35 nm	0	115	138 (66.8)
	1	98	205.8 (103.7)
	24	103	205.7 (90.6)
SiO_2_ 10–20 nm	0	335	321.7 (107.7)
	1	147	223.4 (95.8)
	24	390	312.1 (108.7)
Al_2_O_3_ 13 nm	0	173	191.4 (80)
	1	122	254.2 (106.4)
	24	130	162.2 (78.7)
Al_2_O_3_ 50 nm	0	109	209.9 (106)
	1	77	147.8 (87.6)
	24	74	127.5 (66.1)
Au 5 nm	0	66	106.4 (63.4)
	3	74	167.9 (87.3)
	24	70	124.9 (62.3)
Au 40 nm	0	73	122.3 (67.1)
	3	156	170.6 (44.7)
	24	82	90.8 (20.7)

## Data Availability

Data generated during the study and included in this article will be made available upon request to the corresponding author.
